# Isolation and genomic characterization of selective lytic *Pseudomonas* phage Amjad_SA from a desert urban pond in Riyadh

**DOI:** 10.3389/fmicb.2026.1750744

**Published:** 2026-02-23

**Authors:** Amjad N. Alotaibi, Fahad H. Alqahtani, Nojoud M. ALfayez, Haila M. Alnassar, Abeer H. Alomair, Munirah S. Aleyiydi, Ahmad M. Aldossary, Fahhad D. Alsahli, Abdulmalek T. Algarni, Sarah H. Algahtani, Albatul A. Alshehri, Maha I. Alturki, Fahad M. Alhoshani, Yaser A. Butt, Essam A. Tawfik, Majed S. Nassar

**Affiliations:** 1 Advanced Diagnostics and Therapeutics Institute, King Abdulaziz City for Science and Technology, Riyadh, Saudi Arabia; 2 Advanced Agricultural and Food Technologies Institute, Environment and Sustainability Sector, King Abdulaziz City for Science and Technology, Riyadh, Saudi Arabia; 3 Wellness and Preventive Medicine Institute, Health Sector, King Abdulaziz City for Science and Technology, Riyadh, Saudi Arabia; 4 JEOL Asia Pte Ltd, Singapore, Singapore

**Keywords:** bacteriophage, *Pseudomonas alcaligenes group*, *Pseudomonas* phage Amjad_SA, *Caudoviricetes*, lytic phage, adsorption kinetics, one-step growth, arid aquatic environment

## Abstract

**Introduction:**

Bacteriophages are abundant and diverse components of aquatic ecosystems and contribute to the regulation of bacterial population dynamics. However, *Pseudomonas*-associated tailed bacteriophages from urban aquatic environments within arid regions remain insufficiently characterized.

**Methods:**

We isolated and characterized *Pseudomonas* phage Amjad_SA, a lytic bacteriophage infecting a *Pseudomonas* isolate provisionally assigned to the *Pseudomonas alcaligenes* group (isolate 4L), recovered from an urban artificial pond in Riyadh, Saudi Arabia. The phage was analyzed using transmission electron microscopy (TEM), biological assays (host range, adsorption kinetics, one-step growth, and stability tests), and whole-genome sequencing followed by annotation and phylogenetic analysis.

**Results:**

Phage Amjad_SA exhibited lytic activity against the host isolate and did not infect other tested Gram-negative or Gram-positive bacterial species. TEM revealed an icosahedral head (47.5 ± 1.2 nm) and contractile tail (69.7 ± 1.1 nm), consistent with tailed double-stranded DNA bacteriophages within the class *Caudoviricetes*. Under the experimental conditions tested, the phage retained infectivity following incubation at 2565 °C and across pH 59. Genomic analysis showed a linear double-stranded DNA genome of 45,788 bp with a GC content of 60.4%, encoding 72 predicted open reading frames, including genes associated with genome replication, packaging, host lysis, and virion assembly. A single tRNA-Ser (aga) gene was identified, while a substantial proportion of ORFs were annotated as hypothetical proteins.

**Discussion:**

These findings provide a comprehensive biological and genomic profile of phage Amjad_SA and expand the available reference framework for *Pseudomonas*-infecting tailed bacteriophages recovered from urban aquatic environments within arid regions.

## 1 Introduction

Bacteriophages, or phages, are viruses that infect and lyse bacteria, and can be found in soil, water, and even food. They are now recognized as playing fundamental regulatory roles in microbial ecology and as the most effective candidates for combating antibiotic resistance (AMR; Batinovic et al., 2019; Harada et al., 2018). Since Twort (1915) and d'Herelle (1917) discovered them, phages have been found in every environment inhabited by bacteria, with a ratio of at least 10 phage particles per bacterial cell, making them more diverse than bacteria (Clokie et al., 2011). The global abundance of bacteriophages is estimated to exceed 10^31^, making them the most prevalent life forms on Earth (Bisen et al., 2024; Williamson et al., 2005, 2003). In terrestrial environments, phage concentrations in soil reach approximately 10^8^ particles per gram, and similarly high abundances are reported in aquatic systems, approaching 10^7^ particles per milliliter (Filee et al., 2005; Breitbart et al., 2004). Due to their remarkable abundance and diversity, phages play an essential role in aquatic ecosystems at multiple levels. They contribute to regulating bacterial populations, shape microbial diversity, and influence bacterial evolution and pathogenicity (Canchaya et al., 2003; Miao and Miller, 1999). Additionally, phages contribute to gene flow through horizontal gene transfer, thereby affecting bacterial evolution and adaptation (Canchaya et al., 2003).

There is a resurgence of interest in bacteriophage research as highly specific antibacterial agents, driven largely by the global increase in multidrug-resistant (MDR) bacteria and the limitations of conventional antibiotics (World Health Organization, 2024). Phage intervention presents a highly specific, self-amplifying, and ecologically safe alternative for addressing bacterial infections resistant to chemical antimicrobials (Ali et al., 2023). This specificity also makes them of interest as biocontrol agents for environmental management, as they selectively kill specific bacteria, thereby restoring ecological balance without harming beneficial communities (Harada et al., 2018).

Members of the *Pseudomonadaceae* family, and specifically the genus *Pseudomonas*, are ubiquitous in nature and include both environmental degraders and opportunistic human pathogens. Members of the *Pseudomonas alcaligenes* group are aerobic Gram-negative bacilli distinguished by their metabolic versatility and ability to degrade pollutants such as aromatic hydrocarbons (Pan et al., 2021). Although generally regarded as non-pathogenic, recent clinical reports have associated *P. alcaligenes* and its close relative, *P. paralcaligenes*, with bloodstream and wound infections, and have occasionally reported carbapenem-resistant plasmids (Suzuki et al., 2013; Ono et al., 2023). Because members of the *P. alcaligenes* group are taxonomically and physiologically related to *P. aeruginosa*, they may serve as environmental reservoirs for resistance determinants and virulence-associated genes. Consequently, investigating bacteriophages that target members of the *P. alcaligenes* group contributes to understanding phage–host interactions within environmentally relevant *Pseudomonas* lineages.

Urban, manmade ponds represent engineered microecosystems characterized by variable temperatures, pH levels, salinity, and nutrient inputs (Subramanian, 2024). These systems can harbor diverse microbial communities, including bacteriophages, often subjected to environmental stress shaped by local environmental conditions (2021; Topka et al., 2019). Despite increasing interest in phage ecology, bacteriophage diversity in arid and semi-arid urban aquatic environments remains insufficiently characterized.

Recent developments in genomics have revolutionized the characterization of bacteriophages, thereby enhancing our understanding of their structural organization and evolutionary relationships (Shen and Millard, 2021). High-throughput sequencing has enabled the identification of new genes, including predicted open reading frames (ORFs) with undefined functions that may point to unexplored molecular tools. Notably, the identification of hypothetical proteins in phage genomes reflects the current underrepresentation of closely related phages in public databases and highlights gaps in functional annotations (Sanz-Gaitero et al., 2021). Similarly, analysis of tRNA content can provide insight into phage-host translational interactions. Phages that encode few or no tRNAs depend on the host's translational machinery, consistent with compact genome architectures reported for many lytic bacteriophages (Bailly-Bechet et al., 2007).

Prior research has characterized bacteriophages infecting *Pseudomonas aeruginosa* (Wang et al., 2023) and members of the *P. alcaligenes* group (Ulrich et al., 2024), uncovering substantial genetic diversity. Despite this growing body of work, phage diversity in urban aquatic environments within arid regions remains relatively understudied. Investigating these ecosystems provides an opportunity to identify bacteriophages with distinct structural and genomic features.

In this study, we reported the isolation and genomic characterization of *Pseudomonas* phage Amjad_SA, a lytic bacteriophage recovered from a manmade pond in Riyadh, Saudi Arabia, and active against a *Pseudomonas* isolate assigned to the *P. alcaligenes* group. We combined biological characterization, including host-range assessment, adsorption kinetics, one-step growth analysis, and stability under varying pH and temperature conditions, with genome annotation and phylogenetic analyses. Collectively, these data provide a detailed biological and genomic profile of phage Amjad_SA and contribute to the growing body of knowledge on *Pseudomonas*-infecting tailed bacteriophages isolated from arid urban aquatic environments.

## 2 Materials and methods

### 2.1 Study site description

Several samples were collected from the man-made pond located at the King Abdulaziz City for Science and Technology (KACST), in Riyadh, Saudi Arabia (24 °42′52.0″ N, 46 °38′44.8″ E). The pond covers an area of approximately 3,437.5 m^2^ (length 55 m, width 62.5 m) with an average depth of 0.4 m. During sampling (May–July), ambient temperatures ranged from 30 to 45 °C, and the water pH was 7.0, typical of hot, arid desert environments.

### 2.2 Bacterial strains and growth conditions

The host strain, *P. alcaligenes* BBS 2079, was previously isolated from the same pond. Additional bacterial strains were identified and stored in the Microbiology Laboratory at KACST, and these strains were used to evaluate the phage host range (Table 1). All bacteria were retrieved from −20 °C glycerol stocks, plated onto Luria–Bertani (LB) agar (Invitrogen, Waltham, MA, US), and incubated overnight at 37 °C. For liquid culture preparation, we inoculated single colonies into LB broth (OXOID, Thermo Fisher Scientific, Basingstoke, Hampshire, England, UK) and incubated overnight at 37 °C with shaking at 160 rpm, except for *P. alcaligenes*, which was incubated at 30 °C. Bacterial culture suspensions were standardized to an optical density at 600 nm (OD600) of 1.0 before experiments.

**Table 1:** Bacterial strains used for host-range testing.

**Bacteria species**	**Strain**	**Source**
*Pseudomonas alcaligenes* group	BBS 2079	Manmade pond at KACST
*Pseudomonas aeruginosa*	ATCC 27853	Microbiology lab at KACST
*Pseudomonas aeruginosa*	ATCC BAA-1744	
*Staphylococcus aureus*	ATCC 43300	
*Staphylococcus aureus*	ATCC 29213	
*Escherichia coli*	ATCC 25922	
*Klebsiella pneumoniae*	ATCC BAA-1705	
*Acinetobacter baumannii*	ATCC BAA-747	

## 3 Samples collection and processing

### 3.1 Bacterial samples collection

Samples were collected from the surface water (at a depth of 20 cm) into sterile 50 mL screw-capped conical tubes. They were transported on ice and serially diluted. From each dilution, 0.2 mL was plated on LB agar and incubated at 30 °C overnight. Distinct colonies were subcultured, Gram-stained, and examined microscopically to confirm morphology before storage at 4 °C.

### 3.2 Phage sampling

Six samples, 40 mL each, were collected from the surface of the manmade pond at the designated site using sterile 50 mL screw-capped conical tubes. To remove large soil debris and insoluble particles, the samples were centrifuged at 3,000 rpm for 20 min in 15-mL conical tubes. The collected supernatant was filtered through a 0.45 μm sterile syringe filter. The filtrate (phage lysate) was stored at 4 °C.

## 4 Identification of host bacterium

### 4.1 Bacterial DNA extraction and 16S rRNA amplification

The pure culture of the bacterial strain was streaked onto an LB Agar (Lennox L) plate (Life Technologies, Carlsbad, CA, US) and incubated overnight at 30 °C to yield single isolated colonies. Genomic DNA was extracted from a single bacterial colony using the KingFisher extraction kit (Thermo Fisher Scientific Inc., Waltham, MA, US).

### 4.2 The 16S rRNA amplification

Universal primers for the bacterial 16S rRNA gene were used to identify the bacterial isolate (Weisburg et al., 1991). Polymerase chain reaction (PCR) amplification was carried out in a total volume of 50 μL, which included 1 μL of genomic DNA, 25 μL of Multiplex PCR Master Mix (Qiagen, Hilden, Germany), 2 μL each of the forward primer (AGAGTTTGATCMTGGCTCAG) and reverse primer (GGTTACCTTGTTACGACTT), and 20 μL of nuclease-free water. Amplifications were performed using a Veriti™ 96-Well Thermal Cycler (Applied Biosystems, Foster City, CA, USA). The procedure began with the following cycling program: denaturation at 95 °C for 5 min, followed by 35 cycles of denaturation at 95 °C for 30 s, annealing at 58 °C for 30 s, and extension at 72 °C for 90 s, concluding with a final extension at 72 °C for 7 min. The amplicons were separated on a 1% agarose gel stained with SYBR Safe (Invitrogen, Carlsbad, CA, US) and electrophoresed at 120 V for 30 min. Bands were visualized and analyzed under ultraviolet illumination using a Gel Doc XR+ Imaging System (Bio-Rad, Hercules, CA, US). A single 1,500 bp amplicon was obtained. Subsequently, the amplified DNA was purified using a QIAquick PCR Purification Kit (Qiagen, Hilden, Germany) according to the manufacturer's protocol.

### 4.3 Sequencing of PCR amplifications

DNA sequencing was conducted using the BigDye Direct Cycle Sequencing Kit (Life Technologies, Carlsbad, CA, USA). For the purified PCR product, a sequencing reaction with a total volume of 20 μL was prepared. This reaction combined 3.6 μL of purified DNA, 4 μL of BigDye Direct PCR Master Mix, 1 μL of the reverse 16S rDNA primer (CGGTTACCTTGTTACGACTT), 4 μL of sequencing buffer, and 7.4 μL of nuclease-free water. Thermal cycling was performed as follows: initial denaturation at 96 °C for 1 min, followed by 25 cycles of denaturation at 96 °C for 10 s, annealing at 50 °C for 5 s, and extension at 60 °C for 4 min. Subsequently, the sequencing products were purified using the BigDye XTerminator™ Purification Kit (Applied Biosystems, Foster City, CA, US). Sequencing was performed on an ABI 3730xl DNA Analyzer (Applied Biosystems, Foster City, CA, US) using Sanger sequencing. The resulting 16S rRNA gene sequence was analyzed and compared with reference sequences in the National Center for Biotechnology Information (NCBI) database using BLASTn to determine species-level identity (Ulrich et al., 2024).

## 5 Isolation and enrichment of bacteriophages

In a sterile 15 mL tube, we mixed 3.0 mL of the host strain *P. alcaligenes* overnight culture (OD600 0.5) with equal volumes of each pond sample filtrate and 6 mL of double-strength LB (2 × LB) broth supplemented with 10 mM calcium chloride. The mixture was incubated at 30 °C overnight with shaking at 140 rpm. After incubation, we centrifuged the phage-infected cultures at 5,000 rpm for 20 min to remove excess residual material. We then decanted the supernatant into a sterile tube and filtered it through a 0.45 μm syringe filter to eliminate any remaining bacterial cells. We then added chloroform to the filtrate (1:5), mixed by vortex, and centrifuged at 3,000 rpm for 20 min. After removing the upper aqueous layer, the resulting solution is called the lysate. To verify the presence of the lytic phage in the lysate, a spot assay was conducted on *P. alcaligenes* using the soft agar overlay technique24 to assess the phage's lytic activity against the host. First, 5 mL of semi-solid LB broth (0.7% agar) was mixed with 200 μL of the *P. alcaligenes* culture (OD600 0.5) in a sterile tube. This mixture was then poured onto solid LB agar plates (1.7%) and allowed to solidify. After solidification, 10 μL aliquots of the isolated phage lysate were spotted onto the surface of the semi-solid LB agar in Petri dishes, which were then incubated at 30 °C. Plaque presence was evaluated after overnight incubation. Plates with plaques were considered positive for the lytic phage.

## 6 Bacteriophage isolation by plaque assay

The double agar layer method was used to titrate the phage. First, we mixed 0.1 mL of the phage lysate with 50 μL of the host overnight culture suspension in a sterile 15 mL tube. We then gently vortexed the mixture and incubated it at room temperature for 10 min to promote phage adsorption to the bacterial host. Following this incubation, 5 mL of pre-warmed soft LB agar (0.7% agar) was added to the mixture. The mixture was mixed thoroughly and poured onto an LB agar plate (1.7% agar). This preparation was allowed to set at room temperature for 30 min to solidify, then incubated at 30 °C overnight. After the incubation period, the plates were examined for the presence of plaques, characterized by clear zones on the agar surface. Plates were deemed positive if any plaques were observed. The number of plaques was counted and recorded as plaque-forming units (pfu/mL; Twest and Kropinski, 2009).

## 7 Transmission electron microscopy (TEM) imaging

A high-titer phage lysate (5 × 10^11^ PFU mL^−1^) was added (4 μL) to a copper mesh grid (Carbon Type-B, 200, Tedpella) and subjected to negative staining using 2% (w/v) aqueous uranyl nitrate at pH 5.0. The samples were analyzed using a TEM (JEM-1400, JOEL, Tokyo, Japan) at 100 kV. Images were captured at magnification levels ranging from × 80,000 to × 100,000. Digital recordings were made with a 10.7-megapixel CCD camera (4008 × 2664 pixels, QUEMESA, EMSIS) and iTEM software (Kenny et al., 2004). The morphological characteristics defined by the International Committee on Taxonomy of Viruses (ICTV) were used to evaluate the phenotypic variation of the bacteriophages (Turner et al., 2021; Ackermann, 2011).

## 8 Determining multiplicity of infection (MOI)

The multiplicity of infection (MOI) was calculated based on the absolute numbers of bacteriophage particles and host bacterial cells present at the time of infection. The host culture (*Pseudomonas alcaligenes* group isolate 4L) was adjusted to 2.7 × 10^9^ CFU mL^−1^, as determined by viable plate counts at the time of the experiment. The phage lysate (Amjad_SA) used for infection had a titer of 1.17 × 10^11^ PFU mL^−1^, determined prior to mixing. For infection assays, 100 μL of bacterial suspension was mixed with 100 μL of phage suspension. Because equal volumes were used, the MOI was calculated directly from the ratio of PFU mL^−1^ to CFU mL^−1^, corresponding to an MOI of approximately 40. This MOI was selected to ensure efficient and synchronous infection for adsorption and one-step growth experiments (Kutter and Sulakvelidze, 2004).

## 9 Phage adsorption rate

To determine the adsorption rate of the phage Amjad_SA to the host bacterium, the phage was mixed with *P. alcaligenes* group isolate 4L at the calculated MOI and incubated at 30 °C. For 20 20-min periods, 100 μL of the mixture was collected at 2-min intervals, then diluted with 0.9 mL of LB. The mixture was centrifuged at 5,000 rpm for 15 min. The supernatant containing the unabsorbed free phages was diluted 9-fold. The phage titers were determined using the double-layer agar plate plaque assay. The adsorption rate was measured as the percentage of free, unabsorbed phages in the culture system (Shao and Wang, 2008). The experiment was performed using three independent biological replicates. Mean values and standard deviations were calculated and are reported.

## 10 One-step growth curve

A one-step growth experiment was performed to determine the replication dynamics of phage Amjad_SA. Following adsorption, 7 mL of the host bacterium *P. alcaligenes* group isolate 4L culture was infected with phage Amjad_SA at the optimal MOI. The mixture was then incubated at 30 °C for 5 min. The mixture was then centrifuged at 5,000 rpm for 5 min to remove free, unabsorbed phages. The pellet was resuspended in 7 mL of LB broth and incubated at 30 °C. During the 100-min period, samples were taken at 10-min intervals. Each collected aliquot was diluted and counted using a double-layer agar plate plaque assay (Hyman and Abedon, 2009).

## 11 Phage thermal and pH stability

Phage stability under different pH and temperature conditions was assessed using a plaque assay. For thermal stability, the phage Amjad_SA lysate was divided into six aliquots and incubated at 15, 25, 35, 45, 55, and 65 °C at pH 7 for 6 h. For pH stability, the phage Amjad_SA lysate aliquots were incubated in SM buffer at pH 3, 5, 7, 9, and 11 at 30 °C for 6 h. Following the incubation period, the phages in each aliquot were subsequently titrated by the double agar layer plaque assay (Adams, 1959). However, these assays were performed as single-endpoint measurements and were not designed to assess long-term decay kinetics.

## 12 Host range analysis of bacteriophages

The lytic spectrum was tested against all bacterial strains in Table 1 using spot assays against a panel of Gram-negative and Gram-positive bacterial strains. We prepared double-layer agar plates, each containing a lawn of a different bacterial strain. We spotted 10 μL of the phage Amjad_SA lysate onto each soft-agar lawn of each strain. All assays were incubated overnight at 30 °C, corresponding to the temperature used for phage isolation, and checked for the presence of clear zones (Ackermann, 2011). The temperature was selected to maintain consistent experimental conditions and to avoid potential temperature-dependent effects on phage adsorption and replication. All tested bacterial strains are capable of growth at 30 °C under laboratory conditions. The presence of plaques (clear zones) indicated susceptibility, while the absence of plaques indicated resistance.

The environmentally isolated *Pseudomonas alcaligenes* group strain (isolate 4L) used for phage isolation served as the primary host for host-range testing. Additional *P. alcaligenes* strains were not included due to the limited availability of well-characterized reference isolates accessible during the study. Accordingly, the host-range analysis was designed to assess species-level specificity across the tested bacterial panel rather than intraspecies variation within *P. alcaligenes*.

## 13 Bacteriophage genomic and phylogenetic analysis

### 13.1 Phage DNA extraction and restriction digestion

Phage DNA was extracted from a high-titer phage lysate stock (5.0 × 10^11^ PFU/mL) after bacterial DNA was eliminated with an RNase-free DNase set (Qiagen, Hilden, Germany). Phage DNA was purified using the MagMAX™ DNA Multi-Sample Ultra 2.0 Kit on the KingFisher™ Flex System (Thermo Scientific, Waltham, MA, US). Quantification and assessment of DNA quality were performed using a SpectraMax QuickDrop Spectrophotometer (Molecular Devices, LLC, San Jose, CA, US). To analyze DNA fragment patterns, phage genomic DNA was digested with various restriction endonucleases according to the manufacturer's guidelines (New England Biolabs, Ipswich, MA, US). Four restriction endonucleases were used: BamHI, HindIII, EcoRI, and SmaI. The resulting restriction fragments were resolved by electrophoresis (30 min, 120 V) on a 1% agarose gel (SIGMA, Burlington, MA, US) stained with SYBR Safe (Invitrogen, Carlsbad, CA, US). A DNA molecular weight marker (mi-1Kb DNA Marker; Metabion, Martinsried, Germany) was used to determine the sizes of the DNA fragments.

### 13.2 Phage whole genome sequencing (WGS)

#### 13.2.1 Phage DNA library preparation and sequencing

All phage DNA libraries were prepared according to the manufacturer's instructions using Illumina DNA Prep DNA Flex Library Prep kits (Illumina, San Diego, CA, US). Briefly, phage DNA was tagmented using bead-linked transposomes. The tagmentation reaction was stopped by adding tagmentation stop buffer, followed by post-tagmentation cleanup with washing buffer. The tagmented phage DNA was amplified and barcoded with the Nextera XT Index Kit v2 Set A. The libraries were cleaned and quantified using the Qubit dsDNA High Sensitivity assay kit (Thermo Scientific, Waltham, MA, US). Library size was measured using the Bioanalyzer (Agilent Technologies, Waldbronn, Germany). According to the manufacturer's protocol, each library was normalized to the desired concentration and pooled to a final concentration of 12 pM. The pooled library was spiked with Phix control and sequenced on the Illumina MiSeq platform (San Diego, CA, US). The raw data consisted of 902,797 reads with an average read length of 251 base pairs.

#### 13.2.2 Phage genome sequence assembly and annotation

In the assembly step, we adhere to the guidelines outlined by Shen and Millard 20. We initially performed quality control checks using the FASTQC tool (Andrews, 2010) to assess data quality. Subsequently, raw reads were trimmed of adapter sequences using the Trimmomatic v0.39 tool (Bolger et al., 2014) with SLIDINGWINDOW:4:15 and MINLEN:50, followed by subsampling with the seqtk tool (Shen et al., 2016) to achieve an average coverage of approximately 130. For assembly of the subsampled reads, the SPAdes v3.15.0 tool (Bankevich et al., 2012) was run in careful mode. To identify and correct errors in the assembly, we applied the Pilon tool (Walker et al., 2014). The bbmap.sh script (Bushnell, 2014) was used to calculate coverage statistics for the contigs.

During the annotation step, the Prokka v1.14.6 tool (Seemann, 2014) was used to identify potential proteins, followed by confirmation of these annotations through BLASTp (McEntyre et al., 2002) comparisons. SnapGene v8.2.1 was used to visualize the annotated phage genome (GSL Biotech LLC, 2023). We refined the annotations by conducting BLASTp searches against the NCBI non-redundant protein database. We used an E-value threshold of 1e-10 and considered only query coverage of 70% or higher. We identified conserved domains using the NCBI Conserved Domain Database (Marchler-Bauer et al., 2017). In the updated annotation table for MAJJADAN_00001–00072, we incorporated the results from these BLASTp searches and categorized structural and functional proteins into new categories based on the specific hits identified.

#### 13.2.3 Analysis of tRNA, antibiotic resistance genes, codons, and GC content

Transfer RNA (tRNA) genes were identified using the ARAGORN v1.2.41 (Laslett and Canback, 2004) and the tRNAscan-SE v2.0 tools (Chan et al., 2021) in bacterial mode. To screen for antibiotic resistance genes from the Comprehensive Antibiotic Resistance Database (CARD), we utilized the Resistance Gene Identifier (RGI) v6.0.0 tool (McArthur et al., 2013). Additionally, GC content and start codons were analyzed using an in-house developed Python script.

#### 13.2.4 Phage phylogenetic analysis

To explore genetic similarities with other phages, we performed a Multiple Sequence Alignment (MSA) using MAFFT v7.490 (Katoh et al., 2002), and constructed a phylogenetic tree with IQ-TREE2 (Minh et al., 2020). We included 46 phage whole-genome sequences from GenBank (details in the Supplementary Table S2) and constructed a phylogenetic tree focused on the terminase large subunit, comparing our phage with 14 others (see Supplementary Table S3). Additionally, we conducted a ViPTree analysis of our phage and related phages, using 5,584 phage genomes as reference sequences in ViPTree.

## 14 Results

### 14.1 Isolation and molecular identification of the host bacterium

Four bacterial isolates (designated 4A, 4B, 4C, and 4L) obtained from pond water samples were subjected to near full-length 16S rRNA gene sequencing for taxonomic assignment. BLAST analysis indicated that all isolates belonged to the genus *Pseudomonas*, with isolate 4L showing the highest sequence similarity to *Pseudomonas alcaligenes* strain BBS_2079 and *Pseudomonas aeruginosa* strain BGF-5. Because the 16S rRNA gene is highly conserved among closely related *Pseudomonas* species, species-level resolution could not be achieved with certainty based on this marker alone. Accordingly, isolate 4L was provisionally assigned to the *Pseudomonas alcaligenes* group and was used as the host strain for subsequent phage isolation and propagation.

### 14.2 Bacteriophages: isolation and characterization

**Figure 1:**
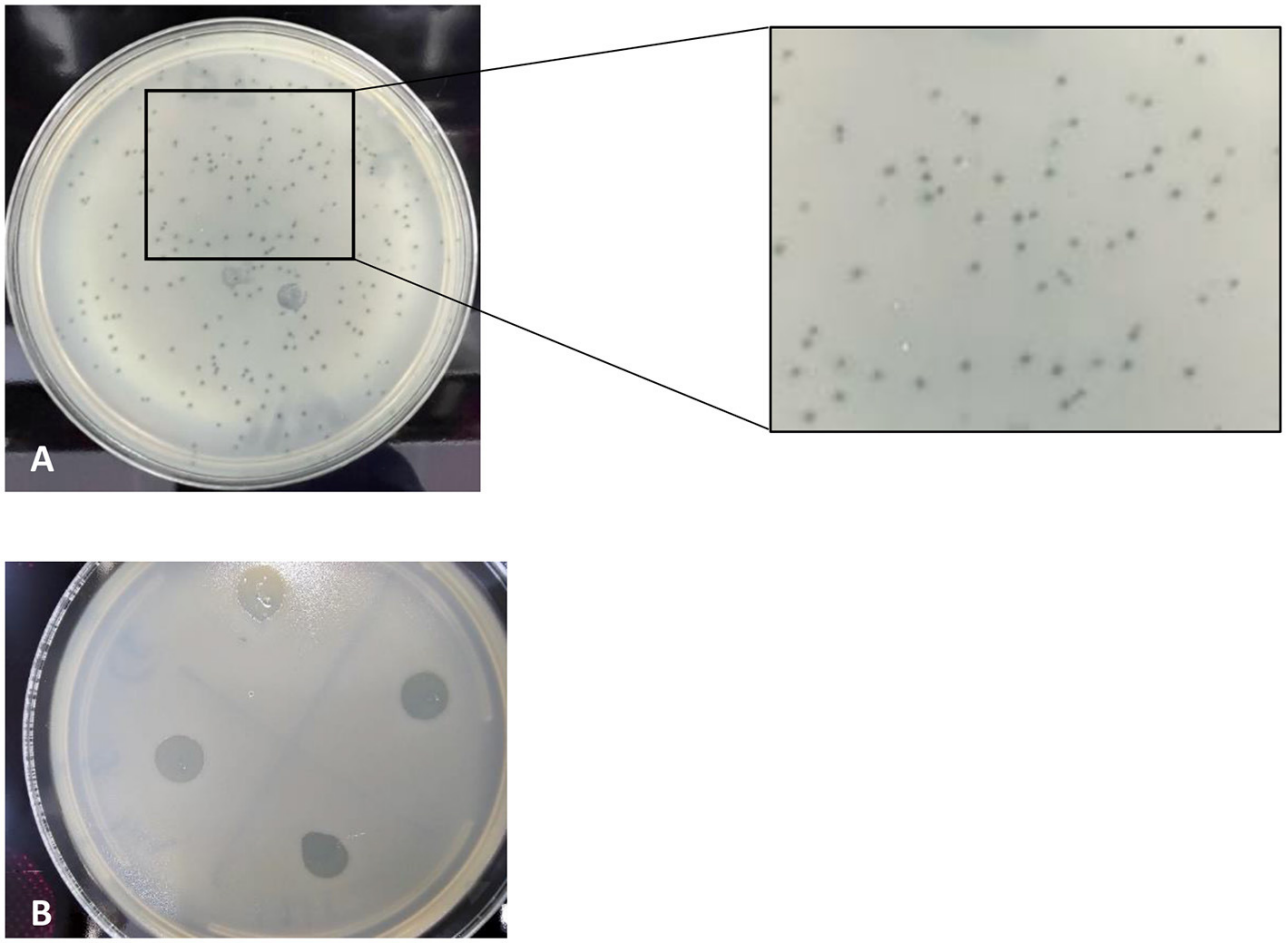
Lytic activity of *Pseudomonas* phage Amjed_SA against the host *P. alcaligenes* bacterium. **(A)** Plaque assay showing clear plaques formed on the host bacterium lawns. **(B)** Spot test demonstrating localized lysis of the isolated phage suspension on the bacterial lawn.

From the same sampling site, six bacteriophages (designated A, J, H, UK, I, and M) were isolated using the *Pseudomonas alcaligenes* group isolate 4L as the host strain. All six isolated phages displayed clear zones on bacterial lawns, indicating a lytic infection phenotype. Plaque assays and spot tests consistently demonstrated localized zones of lysis, confirming productive infection of the host strain (Figure 1). Quantification by the double agar layer method showed that all isolates reached titers on the order of 10^−1^ PFU mL^−1^.

**Figure 2:**
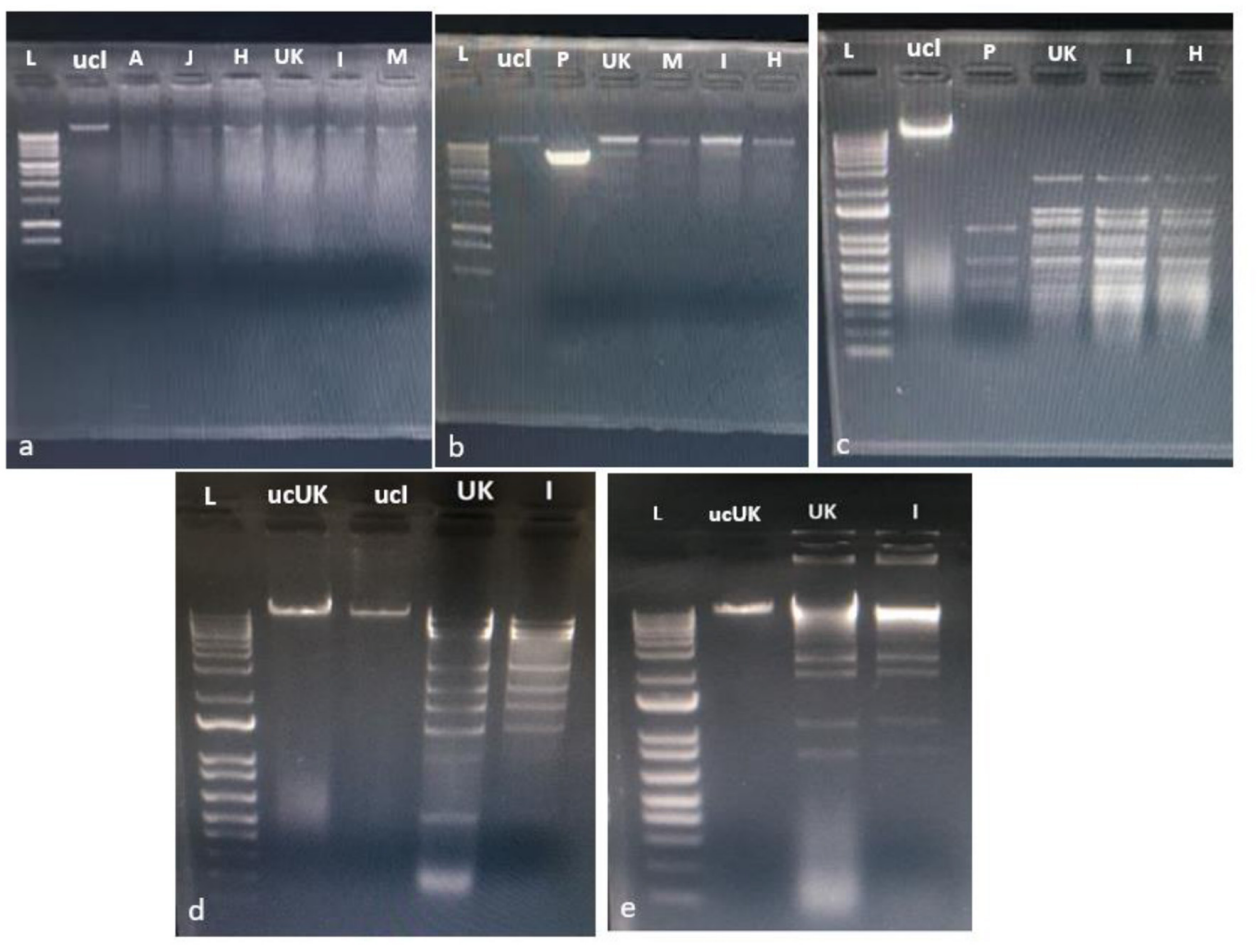
Restriction digestion profile of the five isolated phages: A, J, H, UK, I, and M. Each image represents different phages treated with various restriction enzymes as follows: **(a)** Hind III, **(b)** Bam HI, **(c)** HinF III, **(d)** SmaI, **(e)** EcoRI. Lane-L contains the 1 KB Plus DNA ladder, Lane-P is the control plasmid (pcDNA™5/FRT/TO from Invitrogen), Lane-ucI is the uncut phage (I) DNA, and Lane-ucUK is the uncut phage (UK) DNA.

After extracting DNA from the six isolated phages, we digested each sample with the enzymes HindIII, BamHI, HinFIII, SmaI, and EcoRI. The resulting banding patterns for all six phages were consistently identical (Figure 2), indicating that all isolates from the six sampling sources represent a single phage.

### 14.3 Morphological characterization

**Figure 3:**
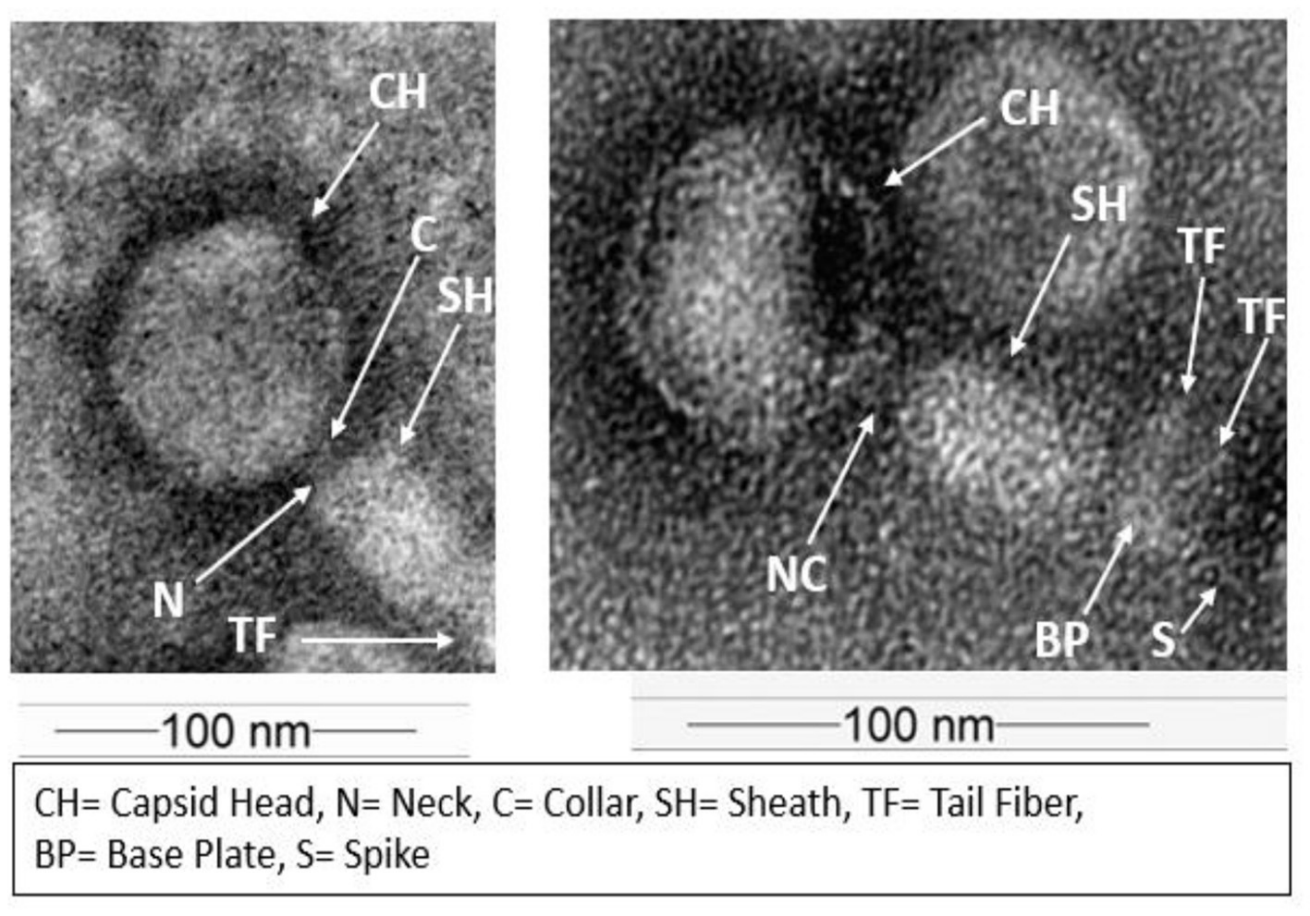
TEM image showing the icosahedral head and contractile tail typical of *Myoviridae* morphology, emphasizing its morphological characteristics.

Negative staining with 2% uranyl nitrate produced high-contrast transmission electron microscopy (TEM) images, enabling clear visualization of the phage structure, including the capsid and tail morphology. TEM analysis of multiple virions revealed a mean icosahedral head diameter of 47.54 ± 1.19 nm and an average contractile tail length of 69.68 ± 1.08 nm, consistent with tailed double-stranded DNA members of bacteriophages within the class *Caudoviricetes* (Figure 3).

### 14.4 Multiplicity of infection and adsorption rate

**Figure 4:**
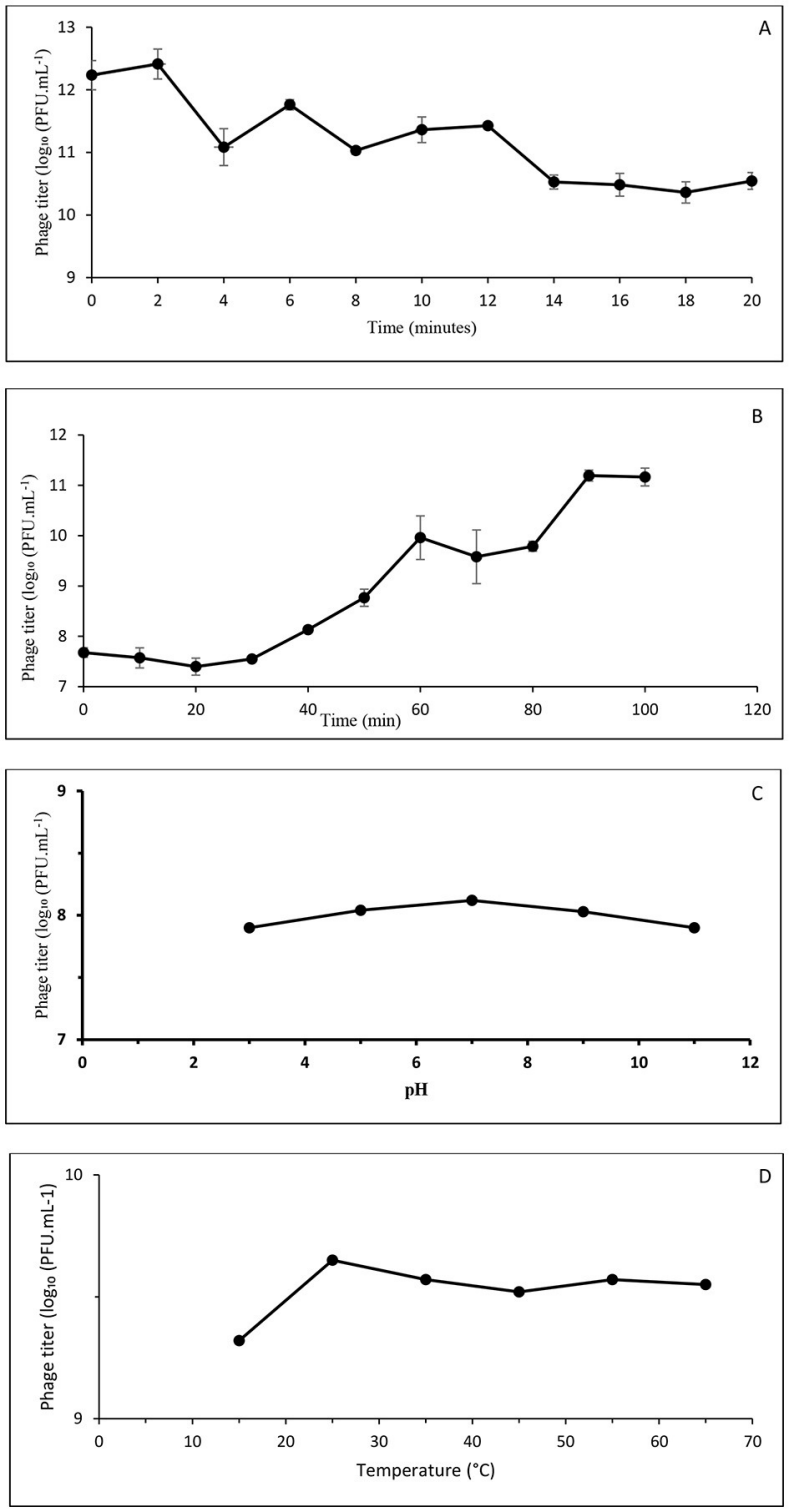
Biological characterization of *Pseudomonas* phage Amjad_SA. **(A)** Adsorption kinetics showing the decline in free phage particles over time following infection. **(B)** One-step growth curve illustrating the latent period, rise phase, and plateau. **(C)** Effect of pH on phage infectivity following 6 h incubation. **(D)** Thermal tolerance of phage Amjad_SA following 6 h incubation at the indicated temperatures. Data represent mean ± standard deviation from three independent biological replicates, where applicable.

The phage adsorption curve showed that phages rapidly adhered to the host bacterium, *P. alcaligenes* group isolate 4L, upon mixing. Across three independent biological replicates, a pronounced rapid decline in the number of free phage particles was observed over time during the first 14 min, corresponding to a reduction of approximately 1.7–2 log10 PFU mL^−1^ relative to initial titers. Free phage concentrations ultimately stabilized between 16 and 20 min when most virions had adsorbed to the bacterial host. Indicating that adsorption had reached an apparent equilibrium by 18 min (Figure 4A).

### 14.5 One-step growth curve

The phage one-step growth curve is a graph that tracks the number of infectious phages over time during a single replication cycle. The one-step growth analysis revealed an initial latent period of approximately 20 min, during which extracellular phage titers remained low. This was followed by a rise phase beginning at approximately 30–40 min post-infection, with phage titers reaching a plateau between 90 and 100 min (Figure 4B).

Based on the mean phage yield at the plateau and the number of host cells present at the time of infection (2.7 × 108 CFU, corresponding to 100 μL of a 2.7 × 10^−1^ CFU mL^−1^ culture), the burst size was calculated to be 590 ± 140 PFU per infected cell (mean ± SD, *n* = 3). These results confirm that the isolated phage is highly productive and lytic.

### 14.6 Effects of pH on phage activity

The observed results in Figure 4C showed that the isolated phage Amjad_SA retained infectivity and stability over the pH range of 5–9. Maximum plaque formation observed at pH 7. Phage exposure to both acidic (pH 3) and alkaline (pH 11) conditions resulted in a slight reduction in viable phage counts. These findings suggest that the isolated phage thrives at neutral pH and can adapt to both high and low pH levels. These results reflect relative tolerance under the tested conditions and do not represent long-term stability.

### 14.7 Phage thermal stability

The temperature-tolerant results showed that the isolated phage remained viable and stable at 25–65 °C, with peak activity at 25 °C (Figure 4D), and that plaque-forming units (PFU) were slightly progressively reduced at higher temperatures. As these assays were conducted as single end-point measurements, they indicate short-term thermal tolerance rather than thermal decay kinetics or long-term environmental stability.

### 14.8 Host-range determination

Host-range testing against the selected eight bacterial strains (Table 1) showed that only the *P. alcaligenes* group was lysed. In contrast, all other tested species, including *Pseudomonas aeruginosa* (two strains), *Staphylococcus aureus* (two strains), *Escherichia coli, Klebsiella pneumoniae*, and *Acinetobacter baumannii*, were resistant. These results demonstrated the strict specificity of the isolated phage for its host bacterium. No plaque formation was observed on any of the tested strains, confirming its narrow host range and indicating that it focused solely on the *P. alcaligenes* group.

## 15 Phage sequencing and identification

### 15.1 Phage genome assembly and annotation

The genome of the *Pseudomonas* phage Amjad_SA is a linear, double-stranded DNA molecule of 45,788 base pairs (bp). It has a GC content of 60.4%, as determined by nucleotide composition (A: 19.4%, C: 31.0%, G: 29.4%, T: 20.2%). Genome assembly resulted in a single contiguous sequence with uniform coverage, and the genomic sequence has been deposited in GenBank under the accession number OP917824.1 (NCBI Taxonomy, 2022).

Automated genome annotation was performed using Prokka, identifying 72 predicted open reading frames (ORFs) with start codon usage of ATG (67), GTG (3), and TTG (2). Of these, 22 ORFs were assigned putative functions based on sequence similarity and conserved domain analysis. Twelve predicted proteins are associated with DNA replication, genome packaging, host cell lysis, and metabolic processes, while 10 ORFs encode structural components of the virion.

The predicted functional repertoire includes genes encoding a DNA helicase, primase, DNA polymerase, and a single-stranded DNA-binding protein. Genes encoding both the large and small terminase subunits were identified, indicating a terminase-mediated DNA packaging strategy. While the presence of these genes is consistent with headful packaging observed in many tailed bacteriophages, the precise genome termini structure could not be experimentally resolved and therefore was not conclusively determined in this study.

The predicted structural module includes genes encoding the major capsid protein, portal protein, tail sheath, tail tube, baseplate wedge, and tail fiber proteins, which are involved in virion assembly and host interaction. The lysis module includes a gene encoding an endolysin with predicted N-acetylmuramidase activity; however, no canonical holin or spanin homologs were detected. In the absence of experimental validation, no alternative lytic mechanism is inferred, and the mode of membrane disruption remains unresolved.

**Figure 5:**
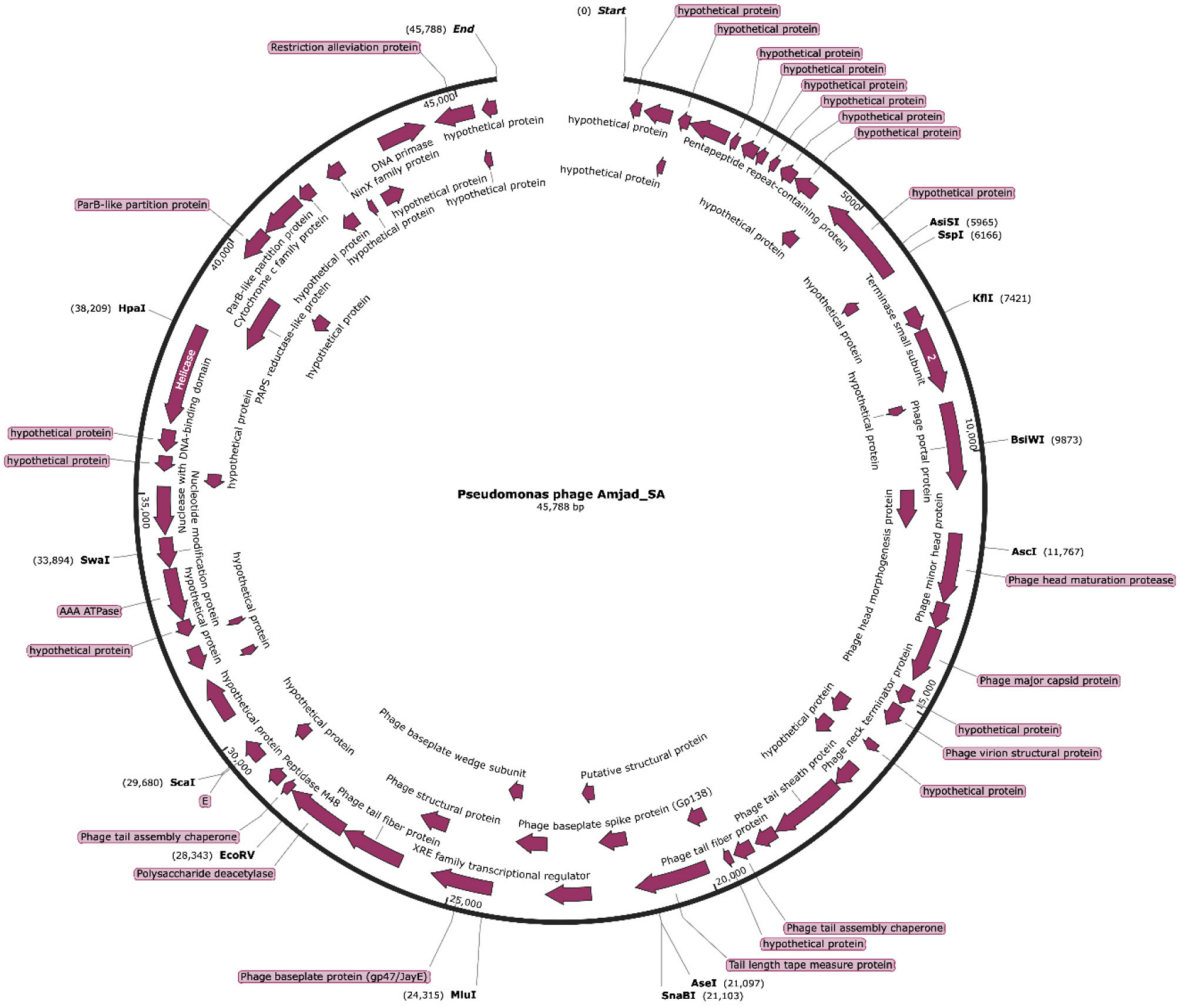
Visualizing the annotated genome using SnapGene (GSL Biotech LLC, 2023).

Approximately 69% of the predicted ORFs encode hypothetical proteins with no confident functional assignment, reflecting the current limitations of phage genome databases and the underrepresentation of closely related reference phages. Genome annotation was performed using established automated pipelines and similarity-based approaches; future re-annotation using updated phage-specific tools such as Pharokka may further refine functional predictions. Annotated ORFs are summarized in Supplementary Tables S1–S4, and a graphical representation of the genome organization is shown in Figure 5.

### 15.2 tRNA and resistance gene analysis

A single tRNA gene corresponding to tRNA-Ser (aga), 667 bp intron, was identified in the genome of phage Amjad_SA. The limited tRNA repertoire suggests reliance on the host translational machinery, a feature commonly observed among lytic bacteriophages with compact genome architectures. In comprehensive genomic screening, no known antibiotic resistance genes were detected associated with determinants or bacterial virulence factors, based on similarity searches against available databases.

### 15.3 Comparative genomics and phylogenetic analysis

**Figure 6:**
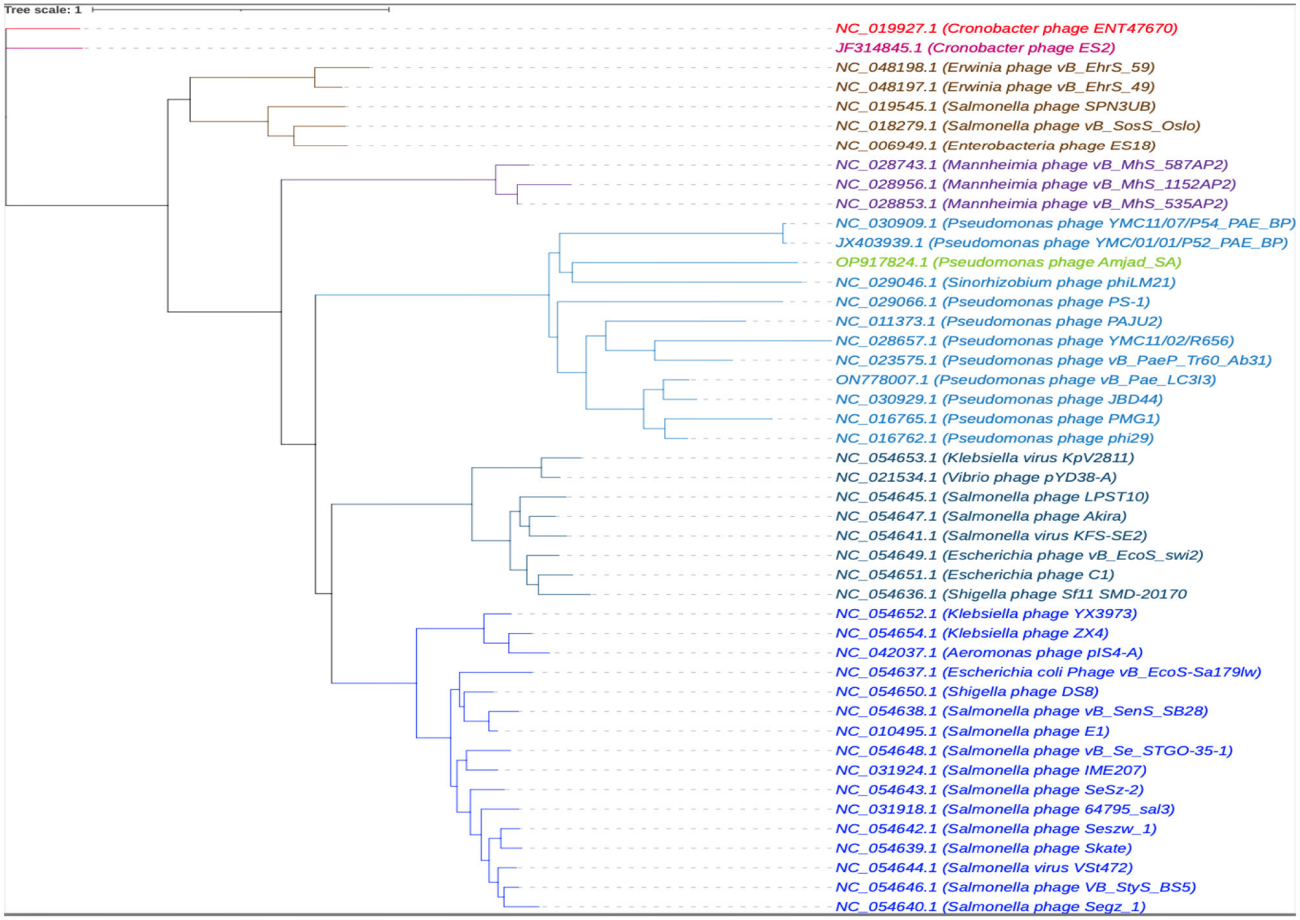
The results of a multiple sequence alignment of 45 whole genome sequences using MAFFT (Seemann, 2014).

To investigate the genetic relationships between phage Amjad_SA and other phages, a multiple sequence alignment (MSA) was performed incorporating 45 whole-genome sequences from GenBank. The resulting phylogenetic tree is shown in Figure 6. The results indicated that phage Amjad_SA clusters closely with phages from the same family, suggesting shared evolutionary traits.

**Figure 7:**
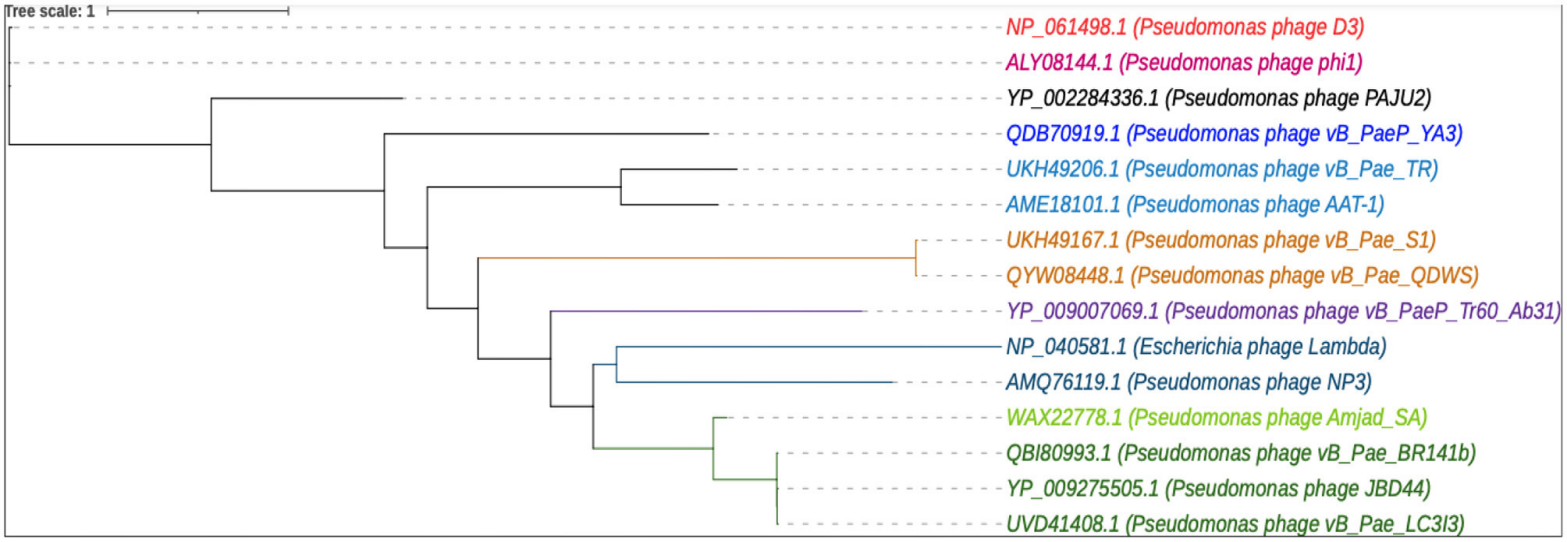
A phylogenetic tree was constructed for the selected phages using their terminase large-subunit sequences.

Furthermore, a focused phylogenetic analysis of the terminase large subunit was conducted, comparing phage Amjad_SA with 14 other phages. The resulting phylogenetic tree is shown in Figure 7. This analysis highlights the conservation of this essential gene across various phage species, reinforcing the evolutionary relationships inferred from the broader genomic analysis.

**Figure 8:**
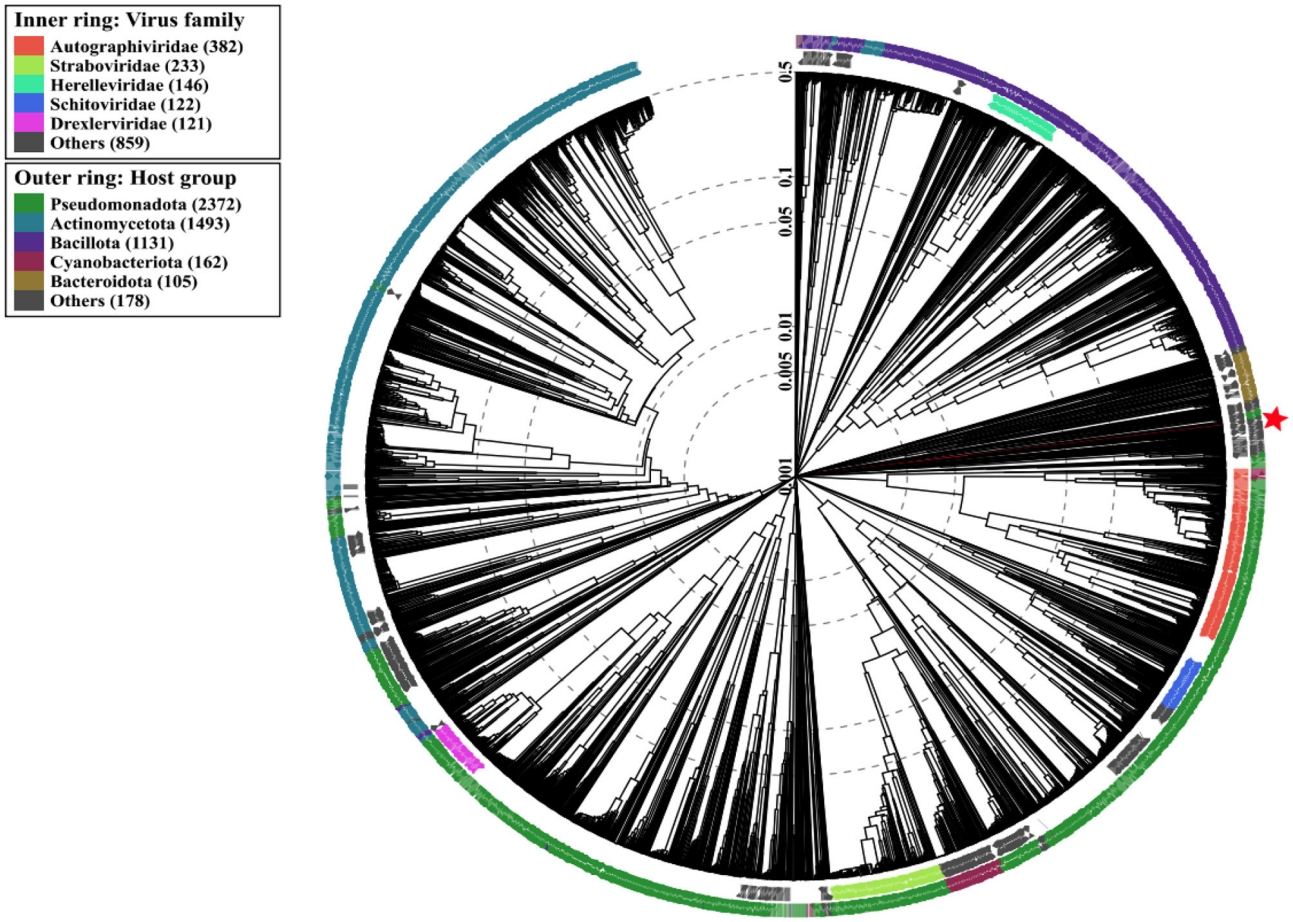
ViPTree (Marchler-Bauer et al., 2017) analysis of our phage Amjad_SA and related phages, with the outer and inner rings representing their host group and virus family, respectively.

To further elucidate the phylogenetic context of the phage Amjad_SA, a ViPTree (Nishimura et al., 2017) analysis was conducted utilizing a comprehensive dataset of 5,584 phage genomes. The results of this analysis are illustrated in Figure 8, which presents a detailed view of the relationships among phages, grouped by host group and viral family. The outer ring represents the host groups, while the inner ring denotes the viral families, providing a clear visual representation of the phylogenetic landscape in which the phage Amjad_SA resides.

**Figure 9:**
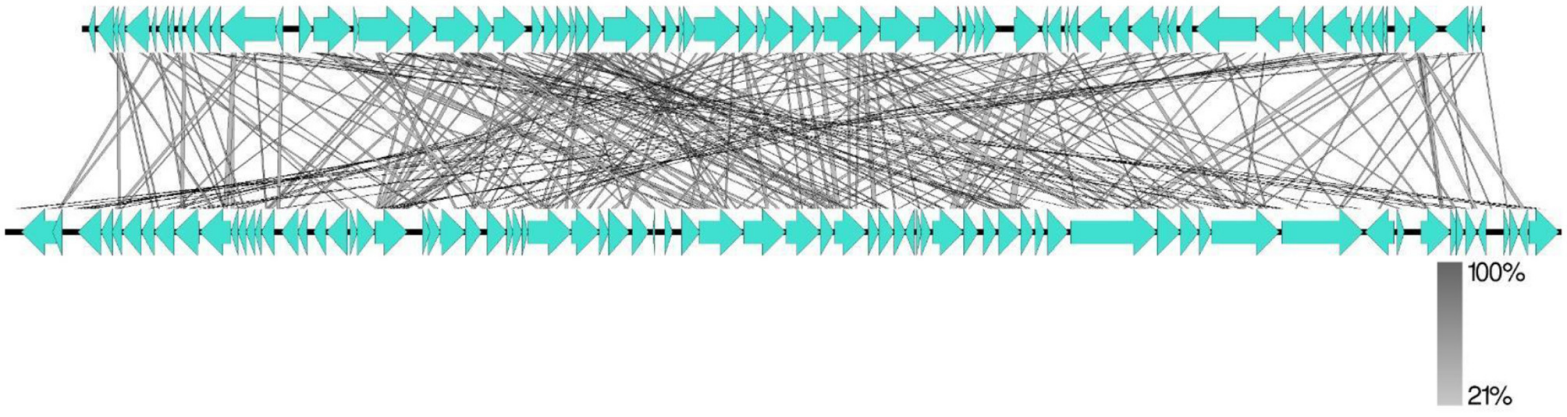
Genome-Wide Comparison of phage Amjad_SA and the *Sinorhizobium* Phage phiLM21 Using Easyfig (Laslett and Canback, 2004).

A genome-wide comparison was performed between *Pseudomonas* phage Amjad_SA and *Sinorhizobium* phage phiLM21, selected based on phylogenetic similarity. This analysis was conducted using Easyfig (Sullivan et al., 2011), and the results are presented in Figure 9. The comparison revealed significant conservation of genomic features, including gene synteny and functional annotations, underscoring the evolutionary relationships between these two phages.

## 16 Discussion

In this study, we report the isolation and characterization of *Pseudomonas* phage Amjad_SA, a lytic bacteriophage recovered from a manmade pond in Riyadh, Saudi Arabia, and active against a *Pseudomonas* isolate assigned to the *P. alcaligenes* group. By integrating biological characterization with genomic and phylogenetic analyses, this work provides a comprehensive profile of phage Amjad_SA and contributes to the expanding dataset of *Pseudomonas*-infecting tailed bacteriophages recovered from urban aquatic environments within arid regions.

Urban artificial ponds in arid regions are exposed to variable physicochemical conditions, including fluctuations in temperature and pH, which can influence microbial community structure. Under the experimental conditions tested, phage Amjad_SA retained infectivity following exposure to temperatures between 25 and 65 °C and across a pH range of 5–9. These results indicate tolerance to the tested temperature and pH ranges; however, longer-term decay kinetics, repeated exposure experiments, and assessment of additional environmental parameters would be required to evaluate environmental persistence or to infer underlying stability mechanisms.

Phage Amjad_SA exhibited a narrow host range, infecting only the *Pseudomonas* isolate used for propagation among the strains examined. Such specificity is consistent with patterns commonly reported for tailed bacteriophages and is generally attributed to host receptor compatibility rather than broad host adaptability. Although host-range restriction has been linked to structural determinants such as tail fibers or baseplate components in other phages, no direct structural or functional evidence was obtained in this study to support specific receptor-binding mechanisms.

Kinetic analyses revealed rapid adsorption, with most virions attaching to host cells within 14 min, and a latent period of approximately 20 min, indicating efficient infection dynamics under the experimental conditions. The estimated burst size (590 ± 140 PFU per infected cell) was comparatively high relative to values reported for several *Pseudomonas*-infecting phages, suggesting productive viral replication. Variation in burst size is known to be influenced by host physiology and experimental parameters, and therefore these kinetic characteristics likely reflect both intrinsic phage properties and host–environment interactions. Collectively, these findings provide insight into the replication efficiency of phage Amjad_SA and underscore its capacity for effective propagation in the tested system.

Genomic analysis showed that Amjad_SA possesses a linear double-stranded DNA genome of 45.8 kb encoding 72 predicted open reading frames. Conserved genes involved in DNA replication, genome packaging, and virion assembly were identified, including terminase subunits, structural proteins, and an endolysin, indicating a typical modular organization of tailed bacteriophages. A substantial proportion of ORFs lacked confident functional annotation, a feature frequently observed in newly characterized bacteriophages and reflective of the limited representation of closely related phage genomes in current databases. These findings contribute additional genomic data to the growing collection of *Pseudomonas*-infecting bacteriophages recovered from urban aquatic environments.

The detection of a single tRNA gene (tRNA-Ser [aga]) suggests a reliance on the host translational machinery, a characteristic commonly reported for lytic phages with compact genomes. Such streamlined genomic architectures are consistent with evolutionary strategies that favor replication efficiency and reduced metabolic burden. Similar patterns have been documented across diverse bacteriophage lineages, in which limited tRNA repertoires support efficient infection cycles (Salmond and Fineran, 2015). Although the functional significance of the identified tRNA gene cannot be fully resolved based on genomic data alone, its presence may reflect adaptive optimization of translational processes during phage replication.

Comparative genomic and phylogenetic analyses placed Amjad_SA within a clade of *Pseudomonas*-infecting tailed bacteriophages, while highlighting genomic distinctions at the level of gene organization and sequence similarity. These differences underscore the genetic diversity present among phages infecting closely related bacterial hosts. However, comparative interpretations were limited to available reference genomes and should be refined as additional *Pseudomonas* phage sequences become available.

Comparative genomic analysis showed that phage Amjad_SA shares sequence similarity with *Sinorhizobium* phage phiLM21 while exhibiting distinct gene organization and variation within structural modules. Phylogenetic analyses based on whole-genome comparisons and terminase large subunit sequences placed Amjad_SA within a distinct clade of tailed bacteriophages infecting *Pseudomonas*. These findings highlight genomic diversity among phages infecting closely related bacterial hosts and underscore the contribution of Amjad_SA to the expanding catalog of *Pseudomonas*-infecting bacteriophages recovered from arid urban aquatic environments.

This study provides a framework for future investigations into the biology and genomics of phage Amjad_SA. Additional work incorporating expanded host panels, receptor-binding studies, proteomic analyses, and functional characterization of hypothetical proteins will be required to better resolve the molecular determinants of host specificity and infection dynamics. Such efforts will contribute to a broader understanding of phage diversity and functional variation among *Pseudomonas*-associated bacteriophages.

Several limitations of this study should be acknowledged. Host identification was based on 16S rRNA gene analysis and did not include whole-genome sequencing or multilocus sequence typing. Environmental parameters beyond temperature and pH were not measured, limiting ecological interpretation. In addition, functional predictions relied primarily on sequence similarity and automated annotation pipelines, and experimental validation of predicted gene functions was not performed. Future studies incorporating expanded host collections, refined genomic annotation approaches, and targeted functional assays will be required to further elucidate the biology of phage Amjad_SA.

## 17 Conclusion

This study presents the isolation and detailed biological and genomic characterization of *Pseudomonas* phage Amjad_SA, a lytic bacteriophage recovered from an urban artificial pond in Riyadh, Saudi Arabia, and active against a *Pseudomonas* isolate assigned to the *P. alcaligenes* group. Morphological analysis by transmission electron microscopy revealed an icosahedral head and a contractile tail, consistent with tailed bacteriophages of the class *Caudoviricetes*. Biological assays demonstrated efficient infection dynamics and a narrow host range under the experimental conditions tested.

Genomic analysis showed that Amjad_SA possesses a linear double-stranded DNA genome of 45,788 bp with a GC content of 60.4%, encoding 72 predicted open reading frames and a single tRNA-Ser (aga) gene. Conserved genes involved in replication, genome packaging, virion assembly, and host lysis were identified, alongside a substantial proportion of hypothetical proteins, reflecting current gaps in functional annotation for *Pseudomonas*-infecting phages. Importantly, no genes associated with known antibiotic resistance determinants or bacterial virulence factors were detected based on sequence similarity searches. Comparative and phylogenetic analyses placed Amjad_SA among related tailed bacteriophages and indicated closest similarity to *Sinorhizobium* phage phiLM21, while also revealing differences in gene organization and sequence similarity that distinguish Amjad_SA from available reference genomes.

Overall, this work expands the available biological and genomic data on *Pseudomonas*-infecting tailed bacteriophages isolated from urban aquatic environments within arid regions. The findings provide a foundation for future studies aimed at refining functional annotation, elucidating phage–host interactions, and further contextualizing bacteriophage diversity associated with *Pseudomonas* species. Collectively, this study delivers a well-resolved biological and genomic dataset for phage Amjad_SA and contributes to the reference framework for *Pseudomonas*-infecting tailed bacteriophages from understudied urban aquatic environments within arid regions.

## Data Availability

The datasets presented in this study can be found in online repositories. The names of the repository/repositories and accession number(s) can be found in the article/Supplementary material.
