# Supplementary material for: Isolation and genomic characterization of selective lytic Pseudomonas phage Amjad_SA from a desert urban pond in Riyadh
Source: Front Microbiol. 2026 Feb 23;17:1750744. doi: 10.3389/fmicb.2026.1750744 (PMC12968170; doi:10.3389/fmicb.2026.1750744)
Supplement: Supplementary file 2 [file Table_2.pdf]

Species	Accession No.	Length
Cronobacter phage ES2	JF314845.1	22,162
Pseudomonas phage YMC/01/01/P52_PAE_BP	JX403939.1	49,381
Enterobacteria phage ES18	NC_006949.1	46,900
Salmonella phage E1	NC_010495.1	45,051
Pseudomonas phage PAJU2	NC_011373.1	46,872
Pseudomonas phage phi297	NC_016762.1	49,135
Pseudomonas phage PMG1	NC_016765.1	54,024
Salmonella phage vB_SosS_Oslo	NC_018279.1	49,116
Salmonella phage SPN3UB	NC_019545.1	47,355
Cronobacter phage ENT47670	NC_019927.1	47,611
Vibrio phage pYD38-A	NC_021534.1	47,552
Pseudomonas phage vB_PaeP_Tr60_Ab31	NC_023575.1	45,550
Pseudomonas phage YMC11/02/R656	NC_028657.1	60,919
Mannheimia phage vB_MhS_587AP2	NC_028743.1	48,594
Mannheimia phage vB_MhS_535AP2	NC_028853.1	50,078
Mannheimia phage vB_MhS_1152AP2	NC_028956.1	52,139
Sinorhizobium phage phiLM21	NC_029046.1	50,827
Pseudomonas phage PS-1	NC_029066.1	48,666
Pseudomonas phage YMC11/07/P54_PAE_BP	NC_030909.1	50,183
Pseudomonas phage JBD44	NC_030929.1	49,033
Salmonella phage 64795_sal3	NC_031918.1	45,342
Salmonella phage IME207	NC_031924.1	47,564
Aeromonas phage pIS4-A	NC_042037.1	47,624
Erwinia phage vB_EhrS_49	NC_048197.1	46,835
Erwinia phage vB_EhrS_59	NC_048198.1	47,116
Shigella phage Sf11 SMD-2017	NC_054636.1	46,454
Escherichia coli Phage vB_EcoS-Sa179lw	NC_054637.1	46,833
Salmonella phage vB_SenS_SB28	NC_054638.1	45,126
Salmonella phage Skate	NC_054639.1	47,393
Salmonella phage Segz_1	NC_054640.1	48,285
Salmonella virus KFS-SE2	NC_054641.1	48,608
Salmonella phage Seszw_1	NC_054642.1	45,881
Salmonella phage SeSz-2	NC_054643.1	45,049
Salmonella virus VSt472	NC_054644.1	46,905
Salmonella phage LPST10	NC_054645.1	47,657
Salmonella phage VB_StyS_BS5	NC_054646.1	47,604
Salmonella phage Akira	NC_054647.1	45,367
Salmonella phage vB_Se_STGO-35-1	NC_054648.1	47,483
Escherichia phage vB_EcoS_swi2	NC_054649.1	47,611
Shigella phage DS8	NC_054650.1	44,605
Escherichia phage C1	NC_054651.1	46,667
Klebsiella phage YX3973	NC_054652.1	46,907

Klebsiella virus KpV2811	NC_054653.1	46,391
Klebsiella phage ZX4	NC_054654.1	45,424
Pseudomonas phage vB_Pae_LC3I3	ON778007.1	49,926
Pseudomonas phage Amjad_SA	OP917824.1	45,788
