# Supplementary material for: Isolation and genomic characterization of selective lytic Pseudomonas phage Amjad_SA from a desert urban pond in Riyadh
Source: Front Microbiol. 2026 Feb 23;17:1750744. doi: 10.3389/fmicb.2026.1750744 (PMC12968170; doi:10.3389/fmicb.2026.1750744)
Supplement: Supplementary file 3 [file Table_3.pdf]

Species	Accession No.	Length
Pseudomonas phage phi1	ALY08144.1	563
Pseudomonas phage NP3	AME18101.1	492
Pseudomonas phage NP3	AMQ76119.1	460
Escherichia phage Lambda	NP_040581.1	641
Pseudomonas phage D3	NP_061498.1	563
Pseudomonas phage vB_Pae_BR141b	QBI80993.1	418
Pseudomonas phage vB_PaeP_YA3	QDB70919.1	488
Pseudomonas phage vB_Pae_QDWS	QYW08448.1	601
Pseudomonas phage vB_Pae_S1	UKH49167.1	601
Pseudomonas phage vB_Pae_TR	UKH49206.1	537
Pseudomonas phage vB_Pae_LC3I3	UVD41408.1	418
Pseudomonas phage Amjad_SA	WAX22778.1	417
Pseudomonas phage PAJU2	YP_002284336.1	505
Pseudomonas phage vB_PaeP_Tr60_Ab31	YP_009007069.1	489
Pseudomonas phage JBD44	YP_009275505.1	418
