# Supplementary material for: Isolation and genomic characterization of selective lytic Pseudomonas phage Amjad_SA from a desert urban pond in Riyadh
Source: Front Microbiol. 2026 Feb 23;17:1750744. doi: 10.3389/fmicb.2026.1750744 (PMC12968170; doi:10.3389/fmicb.2026.1750744)
Supplement: Supplementary file 4 [file Table_4.pdf]

Table S4. Shows the refined list of proteins using BLASTp.

No.	Original Annotation	Amino acids	GenBank Accession	Locus Tag	Proposed Function	Conserved domains
1	hypothetical protein	93	WAX22834.1	MAJJADAN_00072	hypothetical protein	None
2	hypothetical protein	57	WAX22833.1	MAJJADAN_00071	hypothetical protein	None
3	hypothetical protein	258	WAX22832.1	MAJJADAN_00070	Restriction alleviation protein	Lar_restr_allev (pfam14354)
4	hypothetical protein	312	WAX22831.1	MAJJADAN_00069	DNA primase	DnaG (COG0358), Zf-CHC2 (pfam01807)
5	hypothetical protein	163	WAX22830.1	MAJJADAN_00068	hypothetical protein	None
6	hypothetical protein	48	WAX22829.1	MAJJADAN_00067	hypothetical protein	None
7	hypothetical protein	119	WAX22828.1	MAJJADAN_00066	NinX family protein	None
8	hypothetical protein	118	WAX22827.1	MAJJADAN_00065	hypothetical protein	None
9	hypothetical protein	102	WAX22826.1	MAJJADAN_00064	Cytochrome c family protein	None
10	hypothetical protein	296	WAX22825.1	MAJJADAN_00063	ParB-like partition protein	Spo0J (COG1475)
11	hypothetical protein	212	WAX22824.1	MAJJADAN_00062	ParB-like partition protein	IbrB_like (cd16397), ParBc (pfam02195), ParB (smart00470)
12	hypothetical protein	132	WAX22823.1	MAJJADAN_00061	hypothetical protein	None
13	hypothetical protein	402	WAX22822.1	MAJJADAN_00060	PAPS reductase-like protein	YbdN (COG3969), PAPS_reductase-like_YbdN (cd23947)
14	hypothetical protein	652	WAX22821.1	MAJJADAN_00059	Helicase	SSL2 (COG1061), Helicase_C (pfam00271), HELICc (smart00490)
15	hypothetical protein	139	WAX22820.1	MAJJADAN_00058	hypothetical protein	None
16	hypothetical protein	96	WAX22819.1	MAJJADAN_00057	hypothetical protein	None
17	hypothetical protein	100	WAX22818.1	MAJJADAN_00056	hypothetical protein	None
18	hypothetical protein	311	WAX22817.1	MAJJADAN_00055	Nuclease with DNA-binding domain	SAP (pfam02037), SAP (smart00513).
19	hypothetical protein	195	WAX22816.1	MAJJADAN_00054	Nucleotide modification protein	Nmad5 (pfam18757)
20	hypothetical protein	342	WAX22815.1	MAJJADAN_00053	AAA ATPase	AAA (smart00382)
21	hypothetical protein	103	WAX22814.1	MAJJADAN_00052	hypothetical protein	None
22	hypothetical protein	45	WAX22813.1	MAJJADAN_00051	hypothetical protein	None
23	hypothetical protein	149	WAX22812.1	MAJJADAN_00050	hypothetical protein	None
24	hypothetical protein	59	WAX22811.1	MAJJADAN_00049	hypothetical protein	None
25	hypothetical protein	286	WAX22810.1	MAJJADAN_00048	hypothetical protein	None
26	endolysin	142	WAX22809.1	MAJJADAN_00047	endolysin	None
27	hypothetical protein	99	WAX22808.1	MAJJADAN_00046	hypothetical protein	None
28	hypothetical protein	102	WAX22807.1	MAJJADAN_00045	Peptidase M48	None
29	hypothetical protein	59	WAX22806.1	MAJJADAN_00044	Phage tail assembly chaperone	None
30	hypothetical protein	419	WAX22805.1	MAJJADAN_00043	Polysaccharide deacetylase	None
31	hypothetical protein	421	WAX22804.1	MAJJADAN_00042	Phage tail fiber protein	None
32	hypothetical protein	210	WAX22803.1	MAJJADAN_00041	Phage structural protein	None
33	hypothetical protein	404	WAX22802.1	MAJJADAN_00040	Phage baseplate protein (gp47/JayE)	JayE (COG3299)
34	hypothetical protein	116	WAX22801.1	MAJJADAN_00039	Phage baseplate wedge subunit	None
35	hypothetical protein	228	WAX22800.1	MAJJADAN_00038	Phage baseplate spike protein (Gp138)	None
36	hypothetical protein	295	WAX22799.1	MAJJADAN_00037	XRE family transcriptional regulator	None
37	hypothetical protein	99	WAX22798.1	MAJJADAN_00036	Putative structural protein	None
38	hypothetical protein	202	WAX22797.1	MAJJADAN_00035	Phage baseplate protein	None
39	hypothetical protein	479	WAX22796.1	MAJJADAN_00034	Tail length tape measure protein	None
40	hypothetical protein	130	WAX22795.1	MAJJADAN_00033	Phage tail fiber protein	None

41	hypothetical protein	50	WAX22794.1	MAJJADAN_00032	hypothetical protein	None
42	hypothetical protein	128	WAX22793.1	MAJJADAN_00031	Phage tail assembly chaperone	None
43	hypothetical protein	146	WAX22792.1	MAJJADAN_00030	Phage tail fiber protein	None
44	hypothetical protein	495	WAX22791.1	MAJJADAN_00029	Phage tail sheath protein	Pfam11863 (DUF3383)
45	hypothetical protein	168	WAX22790.1	MAJJADAN_00028	Phage neck terminator protein	None
46	hypothetical protein	134	WAX22789.1	MAJJADAN_00027	Phage head protein	None
47	hypothetical protein	62	WAX22788.1	MAJJADAN_00026	hypothetical protein	None
48	hypothetical protein	143	WAX22787.1	MAJJADAN_00025	hypothetical protein	None
49	hypothetical protein	145	WAX22786.1	MAJJADAN_00024	Phage virion structural protein	Pfam13262 (DUF4054)
50	hypothetical protein	117	WAX22785.1	MAJJADAN_00023	hypothetical protein	None
51	hypothetical protein	357	WAX22784.1	MAJJADAN_00022	Phage major capsid protein	None
52	hypothetical protein	168	WAX22783.1	MAJJADAN_00021	Phage minor head protein	None
53	hypothetical protein	450	WAX22782.1	MAJJADAN_00020	Phage head maturation protease	pfam09979 (DUF2213)
54	hypothetical protein	277	WAX22781.1	MAJJADAN_00019	Phage head morphogenesis protein	COG2369, Phage_Mu_F (pfam04233)
55	hypothetical protein	562	WAX22780.1	MAJJADAN_00018	Phage portal protein	COG3567 (DUF1073), pfam06381 (DUF1073)
56	hypothetical protein	58	WAX22779.1	MAJJADAN_00017	hypothetical protein	None
57	Terminase large subunit	417	WAX22778.1	MAJJADAN_00016	Terminase large subunit	XtmB (COG1783)
58	hypothetical protein	146	WAX22777.1	MAJJADAN_00015	Terminase small subunit	None
59	hypothetical protein	81	WAX22776.1	MAJJADAN_00014	hypothetical protein	None
60	hypothetical protein	592	WAX22775.1	MAJJADAN_00013	hypothetical protein	None
61	hypothetical protein	112	WAX22774.1	MAJJADAN_00012	hypothetical protein	None
62	hypothetical protein	160	WAX22773.1	MAJJADAN_00011	hypothetical protein	None
63	hypothetical protein	106	WAX22772.1	MAJJADAN_00010	hypothetical protein	None
64	hypothetical protein	50	WAX22771.1	MAJJADAN_00009	hypothetical protein	None
65	hypothetical protein	61	WAX22770.1	MAJJADAN_00008	hypothetical protein	None
66	hypothetical protein	106	WAX22769.1	MAJJADAN_00007	hypothetical protein	None
67	hypothetical protein	50	WAX22768.1	MAJJADAN_00006	hypothetical protein	None
68	hypothetical protein	262	WAX22767.1	MAJJADAN_00005	Pentapeptide repeat-containing protein	YjbI (COG1357), Pentapeptide (pfam00805)
69	hypothetical protein	72	WAX22766.1	MAJJADAN_00004	hypothetical protein	None
70	hypothetical protein	51	WAX22765.1	MAJJADAN_00003	hypothetical protein	None
71	hypothetical protein	178	WAX22764.1	MAJJADAN_00002	hypothetical protein	None
72	hypothetical protein	68	WAX22763.1	MAJJADAN_00001	hypothetical protein	None
